# Depletion of dendritic cells enhances susceptibility to cell-free infection of human T cell leukemia virus type 1 in CD11c-diphtheria toxin receptor transgenic mice

**DOI:** 10.1186/1742-4690-11-S1-P71

**Published:** 2014-01-07

**Authors:** Saifur Rahman, Sharrón L Manuel, Zafar K Khan, Brian Wigdahl, Edward Acheampong, Frederic Tangy, Pooja Jain

**Affiliations:** 1 Drexel Institute for Biotechnology and Virology Research, and the Department of Microbiology and Immunology, Drexel University College of Medicine, Doylestown, PA, USA

## 

HTLV-1 is associated with two immunologically distinct diseases: HTLV-1-associated myelopathy/tropical spastic paraparesis and adult T cell leukemia. The genesis of these diseases is believed to be associated with the route (mucosa versus blood) and mode (cell-free versus cell-associated) of primary infection as well as the modulation of dendritic cell (DC) functions. To explore the role of DCs during early HTLV-1 infection in vivo, we used a chimeric HTLV-1 with a replaced envelope gene from Moloney murine leukemia virus to allow HTLV-1 to fuse with murine cells, which are generally not susceptible to infection with human retroviruses. We also used a CD11c-diphtheria toxin receptor transgenic mouse model system that permits conditional transient depletion of CD11c(+) DCs. We infected these transgenic mice with HTLV-1 using both cell-free and cell-associated infection routes in the absence and presence of DCs. The ablation of DCs led to an enhanced susceptibility to infection with cell-free but not cell-associated HTLV-1 in both CD4 and non-CD4 fractions, as measured by the proviral load. Infection with cell-free virus in the absence of DCs was also found to have increased levels of Tax mRNA in the non-CD4 fraction. Moreover, depletion of DCs significantly dampened the cellular immune response against both cell-free and cell-associated virus. These results uniquely differentiate the involvement of DCs in early cell-free versus late cell-associated infection of HTLV-1 and highlight a significant aspect of viral immunopathogenesis related to the progression of adult T cell leukemia and HTLV-1-associated myelopathy/tropical spastic paraparesis after the initial infection.

